# Supplementary material for: Splice-Junction-Based Mapping of Alternative Isoforms in the Human Proteome
Source: Cell Rep. Author manuscript; Available in PMC 2020 Jan 15. (PMC6961840; doi:10.1016/j.celrep.2019.11.026)
Supplement: 2 [file NIHMS1546469-supplement-2.zip › DF1/PXD010154/AdrenalGland/AdrenalGland_20_SPATS2_YCQVSSGFIFDLQSNTVLAQGGAFENM.pdf]

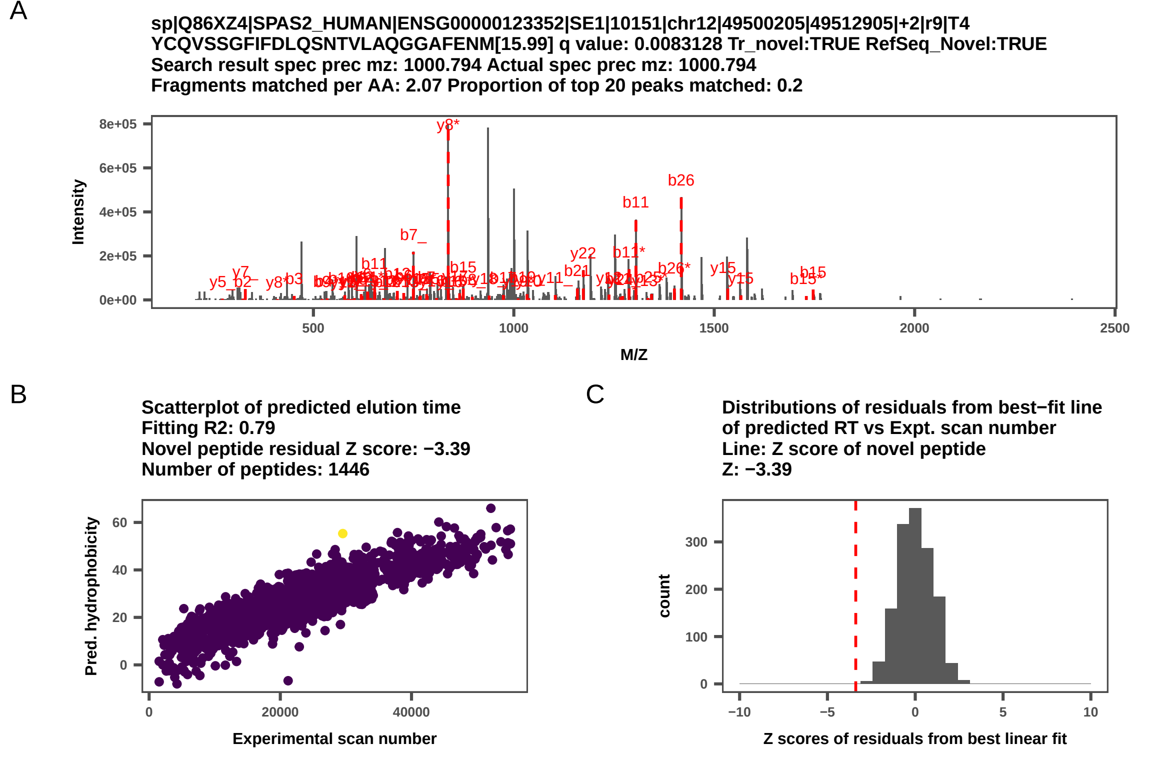
A
sp|Q86XZ4|SPAS2_HUMAN|ENSG00000123352|SE1|10151|chr12|49500205|49512905|+2|r9|T4
YCQVSSGFIFDLQSNTVLAQGGAFENM[15.99] q value: 0.0083128 Tr_novel:TRUE RefSeq_Novel:TRUE
Search result spec prec mz: 1000.794 Actual spec prec mz: 1000.794
Fragments matched per AA: 2.07 Proportion of top 20 peaks matched: 0.2
Intensity
8e+05
6e+05
4e+05
2e+05
0e+00
y8*
b26
b11
b7_
y22
b11*
b26*
y15
b15
b21
b11
y7
y5
b2
y8*
b3
M/Z
500
1000
1500
2000
2500
B
Scatterplot of predicted elution time
Fitting R2: 0.79
Novel peptide residual Z score: −3.39
Number of peptides: 1446
Pred. hydrophobicity
60
40
20
0
Experimental scan number
0
20000
40000
C
Distributions of residuals from best−fit line
of predicted RT vs Expt. scan number
Line: Z score of novel peptide
Z: −3.39
count
300
200
100
0
Z scores of residuals from best linear fit
−10
−5
0
5
10
